# Hemorrhagic Shock and Mitochondria: Pathophysiology and Therapeutic Approaches

**DOI:** 10.3390/ijms26051843

**Published:** 2025-02-21

**Authors:** Nadezda V. Andrianova, Marina I. Buyan, Anna A. Brezgunova, Kseniia S. Cherkesova, Dmitry B. Zorov, Egor Y. Plotnikov

**Affiliations:** 1 A.N. Belozersky Institute of Physico-Chemical Biology, Lomonosov Moscow State University, Moscow 119992, Russia; andrianova@belozersky.msu.ru (N.V.A.); plotnikov@belozersky.msu.ru (E.Y.P.); 2 Faculty of Bioengineering and Bioinformatics, Lomonosov Moscow State University, Moscow 119992, Russia; 3 Faculty of Biology, Lomonosov Moscow State University, Moscow 119992, Russia; 4 V.I. Kulakov National Medical Research Center for Obstetrics, Gynecology and Perinatology, Moscow 117198, Russia

**Keywords:** hemorrhage, hypovolemic shock, multiple organ failure, ischemia, mitochondria, bioenergetics, oxidative stress

## Abstract

Severe injuries and some pathologies associated with massive bleeding, such as maternal hemorrhage, gastrointestinal and perioperative bleeding, and rupture of an aneurysm, often lead to major blood loss and the development of hemorrhagic shock. A sharp decrease in circulating blood volume triggers a vicious cycle of vasoconstriction and coagulopathy leading to ischemia of all internal organs and, in severe decompensated states, ischemia of the brain and heart. The basis of tissue damage and dysfunction in hemorrhagic shock is an interruption in the supply of oxygen and substrates for energy production to the cells, making the mitochondria a source and target of oxidative stress and proapoptotic signaling. Based on these mechanisms, different strategies are proposed to treat the multiple organ failure that occurs in shock. The main direction of such treatment is to provide the cells with a sufficient amount of substrates that utilize oxidative phosphorylation at different stages and increase the efficiency of energy production by the mitochondria. These strategies include restoring the efficiency of mitochondrial complexes, for example, by restoring the nicotinamide adenine dinucleotide (NAD) pool. Another direction is approaches to minimize oxidative stress as well as apoptosis, which are primarily dependent on the mitochondria. There are also a number of other methods to reduce mitochondrial dysfunction and improve the quality of the mitochondrial population. In this review, we consider such strategies for the treatment of hemorrhagic shock and show the promise of therapeutic approaches aimed at restoring the bioenergetic functions of the cell and protecting mitochondria.

## 1. Introduction

Hemorrhagic shock is a life-threatening condition that occurs due to severe blood loss and leads to insufficient tissue perfusion and oxygen supply to vital organs [1]. Hemorrhagic shock happens when the organism loses a significant amount of blood, noting that in an adult, a loss of more than 30% of the circulating blood volume becomes critical. Each year, up to 8% of all deaths are caused by severe injuries and massive bleeding [2], resulting in a total of 1.9 million deaths per year from hemorrhage worldwide [3]. Hemorrhagic shock is responsible for 40% of all deaths due to various injuries and for 27% of deaths during childbirth [4]. The problem of hemorrhagic shock is serious in obstetrics, as postpartum hemorrhage is the most common cause of death associated with pregnancy and childbirth [5]. Death from acute blood loss is not only common in adults but is also observed in pediatric emergency units [6]. An additional problem in children is the inadequate assessment of the severity of hemorrhagic shock and the lack of timely therapy, as they have greater physiological reserves compared to adults and the organism compensates for blood loss in the early stages of shock by maintaining normal blood pressure [7]. Therefore, the clinical signs and symptoms of hemorrhagic shock in children do not appear until more than 20% of the blood volume has been lost, leading to a rapid deterioration of the condition of patients in pediatric wards.

**Figure 1:**
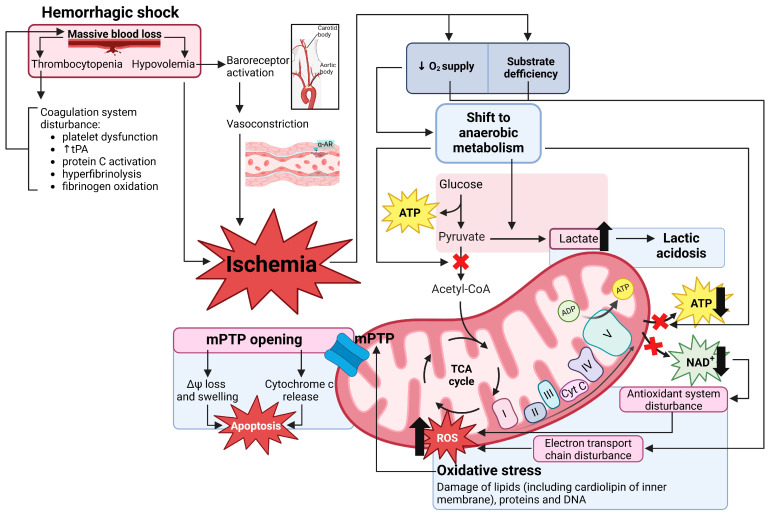
Pathogenesis of hemorrhagic shock. The massive blood loss leads to hypovolemia and thrombocytopenia, resulting in peripheral vasoconstriction and coagulopathy, respectively. The reduction in circulatory volume combined with vasoconstriction leads to tissue ischemia and a subsequent metabolic switch to anaerobic bioenergetics, which manifests itself in lactic acidosis, a decrease in the amount of ATP in the cells, and a disturbed redox balance. These changes result in a dysfunction of mitochondria, the cellular antioxidant defense system, oxidative stress, mPTP opening, and finally, cell death. α-AR—α-adrenoreceptors, ADP—adenosine diphosphate, ATP—adenosine triphosphate, Cyt C—cytochrome c, NAD^+^—nicotinamide adenine dinucleotide, mPTP—mitochondrial permeability transition pore, ROS—reactive oxygen species, and tPA—tissue-type plasminogen activator.

Hemorrhagic shock triggers a complex signaling cascade that leads to damage to vital organs (Figure 1) and multiple organ failure [8,9]. Although the diagnosis of multiple organ failure is made when two or more organs malfunction simultaneously, the adverse changes usually affect the entire organism. Patients who survive multiple organ failure suffer from the chronic diseases it has caused and have a shorter life expectancy [10]. Multiple organ failure does not occur immediately after massive bleeding but may take time to manifest itself [11], with death usually occurring 48 h after the trauma [12]. This means that there is a therapeutic window in which it is potentially possible to reduce the severity of the developing multi-organ failure or even prevent its onset.

Despite significant advances in current medicine, hemorrhagic shock remains one of the leading causes of death and disability worldwide [13]. The development of multi-organ failure in patients with hemorrhagic shock is a predictor of a highly unfavorable outcome. Since most clinically used protocols for the treatment of hemorrhagic shock are not aimed at elucidating the underlying causes of multi-organ failure, novel approaches for the treatment of hemorrhagic shock and associated multi-organ dysfunction are currently being developed. On the other hand, great expectations are placed on the development of approaches that preserve organ cell metabolism and mitochondrial function. In this review, we describe in detail the role of mitochondria in the pathogenesis of hemorrhagic shock and provide an overview of promising therapies aimed at restoring energy metabolism, including the normalization of mitochondrial function.

## 2. Pathogenesis of Hemorrhagic Shock and the Role of Mitochondria

Hemorrhagic shock is often associated with trauma and is a consequence of massive bleeding. A decrease in circulating blood volume and hypovolemia during massive blood loss leads to redistribution of blood flow and other compensatory mechanisms aimed at maintaining blood pressure and supply to critical organs such as the brain and heart [14]. Excessive blood loss (more than 30%) and delayed or insufficient replenishment of circulating blood volume lead to decompensated hemorrhagic shock, resulting in damage to even the brain and myocardium [15]. Severe hemorrhage reduces the effective circulating blood volume, which leads to a decrease in venous return, cardiac output, and organ perfusion pressure. Cerebral circulation and energy metabolism may be well preserved in the compensatory phase, but are impaired in the decompensatory phase [16]. The main cause of death in patients with hemorrhagic shock is the fatal triad, which includes hypothermia, acidosis, and coagulopathy [17].

Impaired blood coagulation is an important factor in the development and exacerbation of hemorrhagic shock, which requires immediate therapeutic intervention [18]. In general, patients with hemorrhagic shock have problems with blood clotting due to decreased platelet count and dysfunction, deficiency of clotting factors, and excessive fibrinolysis [19]. In particular, patients are diagnosed with an increase in the blood concentration of tissue plasminogen activator [20] and activation of protein C [21], which prevents the formation of blood clots and promotes further blood loss. This condition not only exacerbates bleeding but also contributes to the development of disseminated intravascular coagulation, ultimately worsening the patients’ condition [22].

However, the other two components of the fatal triad of hemorrhagic shock are directly related to energy metabolism. Hypothermia occurs as a result of the loss of a large volume of blood, impaired thermoregulation, and reduced heat production due to an insufficient supply of energy substrates. This is facilitated by peripheral vasoconstriction, which serves as a compensatory mechanism to maintain adequate blood pressure under conditions of hypovolemia. Blood loss leads to irritation of vascular receptor zones (including the baroreceptors of the aortic arch and carotid sinus zone), resulting in activation of the sympathetic nervous system, release of catecholamines, and activation of α-adrenergic receptors of smooth muscle cells in the walls of blood vessels, resulting in their constriction. Long-term and significant vasoconstriction leads to redistribution of blood flow, interruption of microcirculation, hypoxia, and lack of nutrient substrates in most organs (kidneys, liver, intestines, pancreas, spleen), skin, and muscles [23], which in the case of decompensated hemorrhagic shock leads to secondary ischemia in the organs and pathological changes in metabolism.

As a result, the third component of the lethal triad develops: metabolic acidosis. It is associated with the conversion of energy metabolism from the aerobic to the anaerobic pathway of adenosine triphosphate (ATP) production under conditions of reduced oxygen and nutrient supply to most tissues and uncompensated loss of intracellular ATP used for essential cellular functions. During hemorrhagic shock, increased glycolysis and decreased lactate utilization, both associated with cellular ATP loss, lead to metabolic acidosis associated with hyperlactatemia, which in itself has adverse effects on organ status [24]. The danger of this condition is explained by the fact that pathologically high lactate production in tissues that are not adapted to lower aerobic energy production at constant energy demand leads to a critical drop in pH to which specific receptors react, activating in response to an acidic shift in pH and triggering the apoptotic cascade of caspases [25]. In addition, acidosis associated with increased lactate leads to impairment of sodium-potassium ATPase and calcium channels. The dysfunction of the Na^+^/K^+^-ATPases leads to sodium retention in the cells and increased potassium excretion. In the cells, the accumulation of hydrogen, sodium, and calcium ions causes hyperosmolarity, which leads to water entering the cytoplasm, swelling of the cells, and ultimately to the organ’s edema. This also leads to a disruption of enzyme activity and stress of the endoplasmic reticulum [26].

In addition to an inadequate supply of oxygen and energy substrates, hemorrhagic shock also leads to mitochondrial dysfunction, which further exacerbates the imbalance between energy supply and energy demand. For example, after hemorrhagic shock, renal mitochondria showed decreased mitochondrial respiratory capacity of all complexes and increased production of reactive oxygen species (ROS) 60 min after resuscitation [27]. Mitochondria in splenocytes after hemorrhage showed significantly reduced oxygen consumption rate, decreased ATP production, decreased mitochondrial membrane potential, and decreased citrate synthase activity [28].

Ultimately, inefficient energy supply to cells leads to a decrease in ATP levels. In particular, it has been shown that 1 h after hemorrhagic shock, a decrease in ATP content is observed in liver and kidney tissue [29]. Similarly, despite the compensatory mechanisms and redistribution of blood flow to the brain and heart during hemorrhagic shock, the content of ATP and creatine phosphate in the myocardium decreased significantly 60 min after resuscitation [30]. In addition to the drop in ATP content due to the transition from oxidative phosphorylation to less efficient energy production pathways, hemorrhagic shock also yields profound changes in the pool of energy-rich compounds and coenzymes. Adenine nucleotides have been shown to be degraded in many tissues, resulting in ATP being converted to adenosine monophosphate (AMP) and then to adenosine and NAD being degraded, ultimately producing nicotinamide and nicotinic acid [31]. Thus, in addition to a significant reduction of NAD^+^ to NADH due to dysfunction of the respiratory complexes and the inability to utilize it, there is a general depletion of the NAD^+^/NADH pool, making it difficult to restore energy metabolism even after normalization of the blood supply [32]. NAD^+^/NADH not only play a key role in energy metabolism, but are also required for the maintenance of the cell’s oxidative and antioxidant systems, calcium homeostasis, regulation of gene expression, post-translational modifications of proteins, etc. [33], so their loss can be detrimental to energy metabolism.

Restoration of blood flow and resumption of oxygen and nutrient supply to cells after a period of hypoxia can lead to excessive formation of ROS, which damages macromolecules in cytosol and organelles and causes further organ dysfunction [34]. During ischemia, the mitochondrial electron transport chain complexes are disrupted and the levels of coenzymes and heme in the respiratory chain are downregulated. The further reintroduction of oxygen and energy substrates can lead to a one-electron leak from Complex I and Complex III to a dioxygen-forming superoxide molecule (O_2_^·−^) and then hydrogen peroxide [35]. During reperfusion, a process called reverse electron transfer can also take place, in which electrons flow uphill from Complex II to Complex I, resulting in significant ROS production at Complex I [36]. Another mechanism that causes ROS production is the accumulation of succinate during the ischemic phase, which leads to its rapid oxidation at the reperfusion phase, reverse electron transfer, and a burst of ROS production at Complex I [37]. The rapid restoration of oxygen can also open the mitochondrial permeability transition (mPTP) with the proper collapse of the mitochondrial membrane potential, inducing a burst of ROS production [38]. The alarming rise in ROS in cells, resulting from an imbalance between the activity of cellular antioxidant systems and ROS generation during reperfusion leads to oxidative stress [39]. Low-level oxidative stress and dysfunction of the electron transport chain trigger a cascade of ROS formation induced by mPTP associated with the disruption of the barrier function of the mitochondrial inner membrane, ultimately causing mitochondrial swelling, inner membrane depolarization, and subsequent release of cytochrome c culminating in cell death [40]. As a result of all the events described, exposed cells die by necrosis and apoptosis, both in the phase of decompensated hemorrhagic shock and after restoration of circulating volume and resumption of tissue perfusion. Oxidative stress also exacerbates the development of coagulopathy in patients with hemorrhagic shock, as fibrinogen is very sensitive to oxidation, reducing its ability to form a fibrin clot [41].

Damaged and dying cells, especially in the case of necrosis, release damage-associated molecular patterns (DAMPs) into the intracellular environment, which include glycosaminoglycans (syndecans, hyaluronic acid, and heparan sulfate), some nuclear proteins (e.g., histones, HMGB1, and HMGN1), nuclear DNA, some cytosolic proteins (e.g., heat shock proteins, S100 proteins, and F-actin), as well as calreticulin and components of cell-derived microvesicles [42,43]. There are also mitochondrial DAMPs, which include mtDNA, ATP, cardiolipin, mitochondrial transcription factor A, N-formyl peptide, and succinate [44,45]. Any tissue or organ damaged by hemorrhagic shock is a potential source of DAMPs [46]. DAMPs trigger the cascade of innate immune responses, including those activated by Toll-like receptors and nucleotide-binding and oligomerization domain (NOD)-like receptors, which transmit signals locally or systemically via the bloodstream leading to activation of immune cells and the complement system [47]. The release of DAMP signaling molecules ultimately leads to a systemic inflammatory syndrome, which further stimulates the production of inflammatory mediators and worsens the condition of the organism [48,49]. In patients with severe trauma, mitochondrial DAMPs have been shown to play a key role in activating the inflammatory response [50]. Ultimately, “sterile inflammation” develops, in particular, systemic inflammatory response syndrome and compensatory anti-inflammatory response syndrome [51].

## 3. Therapeutic Approaches Against Hemorrhagic Shock

### 3.1. Resuscitative Fluid Therapy

The approaches used in clinical practice to treat hemorrhagic shock are based on infusion and blood replacement therapy [52]. Depending on the severity of the blood loss and the time that has elapsed since the onset of bleeding, different strategies are used [53]. Thus, in modern clinical recommendations, in the case of severe blood loss, i.e., about 30–40% of the circulating blood, the implementation of a massive transfusion protocol of a crystalloid solution with blood products is recommended, while in the case of blood loss of 15 to 30%, an infusion of a crystalloid solution is performed until the systolic pressure is normalized, followed by a blood transfusion [54]. Standard resuscitation solutions can optionally be supplemented with hypertonic solutions, oxygen-carrying blood substitutes such as synthetic fluorocarbon-based oxygen carriers, and stroma-free cross-linked human or non-human hemoglobin products [55].

However, most infusion procedures are associated with a number of side effects due to the discrepancy between the resuscitation solutions and all parameters of real blood. Thus, when restoring the lost blood volume with substitutes, it is necessary to monitor the electrolyte balance so as not to miss the development of hypocalcemia and hyperkalemia [56]. Another unresolved problem of massive transfusion in cases of large blood loss is the negative side effects due to the large fluctuations in the osmolarity of the extracellular fluids. Crystalloid solutions have been shown to be nephrotoxic, which combined with the ischemia caused by impaired perfusion of renal tissue, leads to fatal renal damage [57]. In addition, massive blood replacement can provoke the development of hemolysis, which leads to an increase in free iron ions in the bloodstream, increased oxidative stress, and damage to the vascular endothelium [58].

### 3.2. Coagulopathy Treatment

Coagulopathy, which occurs in patients with hemorrhagic shock, is associated with possible organ failure and a 4-fold increase in mortality and therefore requires immediate therapy [59]. In hemorrhagic shock, thrombocytopenia is observed due to significant blood loss, which can be exacerbated by transfusion therapy with blood-substituting fluids. To avoid this, it is recommended to infuse fresh frozen plasma with platelet concentrate after replenishing the circulating blood volume with crystalloid solutions [60]. In addition, compounds such as aprotinin, tranexamic acid, epsilon-aminocaproic acid, and aminomethylbenzoic acid are used as antifibrinolytic agents, which significantly reduce the development of coagulopathy in patients [61,62].

### 3.3. Support of Energy Metabolism and Mitochondrial Function

**Figure 2:**
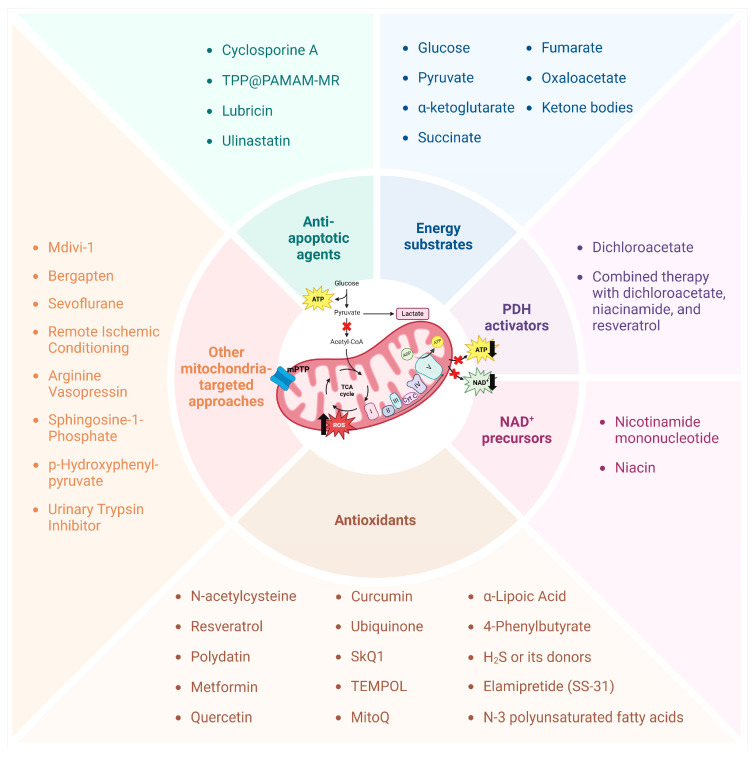
Strategies proposed in clinical and experimental studies to maintain metabolism and mitochondrial function during hemorrhagic shock and after resuscitation. The main principles of action and the substances used to implement these strategies are presented.

Despite the undisputed importance of transfusion therapy and correction of coagulopathy, these approaches provide only temporary support of critical functions in hemorrhagic shock without affecting the molecular processes occurring in the damaged organs. These processes are primarily associated with a lack of energy substrates and oxygen and are in many ways similar to the mechanisms of ischemic injury [63]. Existing replacement infusion–transfusion therapy administered during hemorrhage cannot fully compensate for this deficit [64]. For this reason, novel treatment approaches based on the maintenance of cellular metabolism and the prevention of bioenergetic collapse are being actively developed and implemented [65]. In this section, we review the already clinically used and experimental strategies to maintain metabolism and mitochondrial function during hemorrhagic shock and after resuscitation (Figure 2).

#### 3.3.1. Replenishment of the Energy Substrates

The mechanisms of metabolic disorders described above are primarily based on an inefficiency of oxidative phosphorylation pathways and mitochondrial function. Therefore, approaches aimed at maintaining an adequate bioenergetic supply assume that the supply of the cells is increased primarily with intermediates of the tricarboxylic acid cycle and substrates that can be rapidly oxidized by the mitochondria. Indeed, pyruvate administration has been shown to be effective in experimental models of hemorrhagic shock, improving systemic and cerebral oxygen delivery and consumption, cerebral blood flow, and preventing the development of lactic acidosis [66]. Administration of pyruvate 30 min after the onset of hemorrhagic shock reduced animal mortality, delayed cardiovascular decompensation, reduced neuronal de-energization, and significantly improved metabolic and functional brain status [67]. Metabolic changes included an increase in ATP production in the cerebral cortex, a decrease in the accumulation of ATP breakdown products, and the lactate/pyruvate ratio. Hepatoprotective effects of pyruvate as part of resuscitation therapy have been demonstrated, decreasing the level of damage markers, improving mitochondrial respiration in liver tissue, and also decreasing inducible nitric oxide synthase levels, fragmentation of poly-ADP ribose polymerase, and cytochrome c release [68]. Resuscitation with Ringer’s solution with pyruvate significantly improved serum markers of organ dysfunction in rats, completely corrected severe lactic acidosis 1 h after resuscitation, and significantly lowered blood lactate levels [69].

Administration of energy substrates such as glucose, fumarate, α-ketoglutarate, and oxaloacetate, also as part of combination therapy, increased animal survival and prevented the development of acidosis in hemorrhagic shock in rabbits [70]. However, administration of the tricarboxylic acid cycle metabolite succinate has a number of limitations, as excess succinate may play a negative role in the early stages of hemorrhagic shock. It is known that hemorrhagic shock, similar to ischemia, leads to an accumulation of succinate in cells as its utilization by the mitochondrial Complex II is impaired [71,72]. As a result, during reperfusion, excess succinate is rapidly consumed by the respiratory chain, generating a large number of active oxygen species [72,73,74]. Inhibition of succinate dehydrogenase, which is responsible for the accumulation of succinate in the first (ischemic) phase of shock, e.g., with dimethylmalonate, is an effective strategy to prevent cell damage [75], which reduces ROS production [72]. However, the administration of succinate has a positive therapeutic effect after compensation of hypovolemia and restoration of tissue oxygenation. In patients treated with succinate-containing drugs for ulcerative gastroduodenal bleeding, it has been observed that the extent of specific disturbances of cell metabolism under critical conditions, such as tissue hypoxia, mitochondrial dysfunction, free radical oxidation, lipid peroxidation, and imbalance of the antioxidant system, is significantly reduced [76]. It should be noted that recent studies exploring the mechanisms of ROS generation in mitochondria indicate the possibility of a more targeted treatment of pathological oxidative stress [77]. In particular, it has been found that the excessive generation of ROS at Complex I is associated with pathological oxidative stress and that lowering this production could be beneficial [78]. In contrast, ROS production in Complex III is likely to contribute more to the regulatory and signaling effects of ROS and, conversely, may serve as a means of tissue adaptation to oxidative stress. This should also be considered in the development of tissue protection strategies for hemorrhagic shock.

Since conventional pathways of cellular energy supply may be ineffective in hemorrhagic shock, alternative metabolic substrates such as ketone bodies, which can be directly converted to acetyl-CoA and further enter the tricarboxylic acid cycle, can be used to rapidly replenish oxidative phosphorylation [79]. The administration of beta-hydroxybutyrate in hemorrhagic shock had a positive effect on the partial pressure of oxygen and carbon dioxide and the amount of lactate in the blood [68]. Combination therapy of beta-hydroxybutyrate with melatonin, an antioxidant and free radical scavenger, reduced mortality, transiently increased mean arterial pressure, lowered base deficit, and increased oxygen consumption and serum levels of succinate, 2-oxovalerate, and adipate [80,81,82].

#### 3.3.2. NAD^+^ Precursors

A promising therapeutic approach is the maintenance of normal NAD levels, which ensures the functioning of the respiratory chain [32]. In addition, NAD^+^ is a co-substrate for sirtuins, in particular, SIRT1, which is associated with many pro-survival cascades [83]. A beneficial effect on animal survival of exogenous nicotinamide mononucleotide (NMN) or niacin, which are precursors of NAD^+^, has been demonstrated when administered before or during resuscitation from hemorrhagic shock. In both the liver and kidneys, NMN prevented mitochondrial dysfunction and preserved Complex I-dependent mitochondrial respiration, which was impaired during hemorrhage. In the kidneys, but not in the liver, NMN was sufficient to prevent ATP loss after shock and resuscitation and also reduced the severity of the inflammatory response [29]. Oral administration of niacin in conjunction with whole blood resuscitation improved hemorrhagic shock-induced acute lung injury, decreased malondialdehyde levels in the lungs, downregulated the NF-κB signaling pathway, and suppressed the inflammatory response [84].

#### 3.3.3. Increase in Pyruvate Dehydrogenase Activity

A therapeutic target for alleviating the consequences of hemorrhage may be the pyruvate dehydrogenase complex (PDC), the activity of which decreases sharply upon reperfusion [85]. The activity of the complex is regulated by secondary modifications, primarily through its phosphorylation by PDH kinase. Usually, PDH kinase inactivates the complex at high acetyl-CoA/CoA, NADH/NAD^+^, and ATP/ADP ratios, which is necessary to prevent glucose utilization under conditions of switching energy metabolism to fatty acid oxidation. Reactivation of PDH occurs by dephosphorylation by a specific phosphatase, which is stimulated by NAD^+^ and calcium and inhibited by NADH [85,86]. The activity of PDH kinase under hypoxic conditions is explained by levels of NADH and acetyl-CoA [87]. In the post-ischemic period, PDH activity can be increased by inhibiting PDH kinase, for example, with dichloroacetate [88]. Indeed, dichloroacetate has shown its efficacy in models of hemorrhagic shock, improving both hemodynamic parameters and mitochondrial status [89]. Combination therapy with dichloroacetate, niacinamide, and resveratrol also improved survival rates after hemorrhagic shock and significantly reduced levels of proinflammatory interleukins [90]. Moreover, dichloroacetate therapy is already used in clinical practice for the treatment of lactic acidosis and has shown its high efficacy in alleviating acute, chronically acquired, and inherited forms of lactic acidosis [91].

#### 3.3.4. Antioxidants, Including Mitochondria-Targeted

Since the pathogenesis of hemorrhagic shock is associated with the development of oxidative stress, the use of antioxidants may be a promising approach for therapy. The use of the antioxidant N-acetylcysteine after hemorrhage and fluid resuscitation led to a significant decrease in the level of thiobarbituric acid reactive substances, superoxide radicals, and NO in kidney tissue, which indicates a decrease in oxidative stress, as well as a reduction in the expression of Bax, cytochrome c, and caspase-3, suggesting a reduction in hemorrhagic shock-induced apoptosis in the kidney [92]. Similarly, in hemorrhage aggravated by immersion in seawater, treatment with N-acetylcysteine counteracted acidosis, promoted normalization of pCO_2_ and pO_2_, improved coagulation, and protected the kidneys, liver, and heart, as evidenced by reductions in urea, creatinine, AST, ALT, and troponin T levels [93].

Another way to reduce reperfusion stress is to use the natural polyphenol resveratrol, which exhibits antioxidant properties. Administration of resveratrol during resuscitation of hemorrhagic shock restored oxidative phosphorylation in renal mitochondria and decreased oxidative stress [27,94]. Resveratrol also reduced hemorrhagic shock-induced liver injury [94] and decreased the production of DAMPs and the extent of oxidative stress [95]. An additional mechanism that may enhance the antioxidant effect of resveratrol could be the activation of SIRT1 [96]. Resveratrol also exerts anti-inflammatory effects mediated by activation of the AKT signaling pathway [97,98]. Polydatin, which is structurally similar to resveratrol, can also prevent mitochondrial dysfunction and exhibits antioxidant properties. Polydatin treatment protects neurons in hemorrhagic shock by maintaining normal mitochondrial morphology, maintaining normal Δψ levels, and preventing mPTP opening [99]. In addition, polydatin neutralized the decrease in ATP levels and reduced lipid peroxidation. Polydatin also had a positive effect on rat hepatocytes by preventing mPTP opening and Δψ drop as well as reducing ROS production and lipid peroxidation and increasing the level of reduced glutathione [100].

Treatment with the antidiabetic drug metformin, which may have antioxidant properties, reduced lung and liver damage in mice exposed to hemorrhagic shock. In addition, metformin therapy significantly reduced myeloperoxidase activity in the lungs and liver, increased ATP levels in the liver and heart, and reduced lipid peroxidation. The beneficial effect of metformin was also maintained in AMPKα1 KO mice, which may indicate that the effects are realized through antioxidant properties rather than AMPKα1 signaling pathways [101]. The use of the flavonoid quercetin, which has antioxidant, antiplatelet, anti-inflammatory, vasodilatory, and antihypertensive effects, is also being considered as a therapy for hemorrhagic shock. Its administration has been shown to reduce pathological changes in the lungs [102]. The widely studied antioxidant curcumin significantly reduced vascular permeability in a model of hemorrhagic shock by reducing oxidative stress in endothelial cells [103]. Another known antioxidant that can be used as an additional component of infusion therapy is coenzyme Q10. It has been shown that the administration of coenzyme Q10 after hemorrhagic shock reduced the production of mitochondrial superoxide by leukocytes and of hydrogen peroxide in the diaphragmatic tissue and also prevented apoptosis of cells in the lungs, diaphragm, kidneys, and heart [104].

In addition, the possibility of using mitochondria-targeted antioxidants, which have been developed over the last two decades, such as SkQ1, are considered very promising antioxidants. A whole range of protective effects of SkQ1 has also been demonstrated in hemorrhagic shock: a decrease in ROS in cardiac muscle tissue, restoration of mitochondrial structure and function, and a decrease in the expression of the proinflammatory cytokines TNF-α, IL-6, and MCP-1 [105]. Another group of mitochondria-targeted antioxidants that have demonstrated efficacy in attenuating the effects of hemorrhage are compounds based on stable nitroxide radicals, such as 4-hydroxy-2,2,6,6-tetramethylpiperidine-1-oxyl (TEMPOL) conjugated to the mitochondria-targeted portion of gramicidin S [106,107]. Promising mitochondria-targeted antioxidants are the Szeto–Schiller peptides (SS-peptides), which scavenge ROS and inhibit lipid oxidation of mitochondrial membranes, effectively downregulating the induction of apoptosis and preventing tissue damage [108]. Resuscitation fluid containing elamipretide (SS-31) had a positive effect on serum creatinine level, troponin and interleukin-6 concentrations, and improved histological changes in the liver and duodenum [109]. It should be noted that another mitochondria-targeted antioxidant, MitoQ, reduces mortality and inflammation in hemorrhagic shock, but also increases lipid peroxidation [110]. Therefore, it is necessary to be cautious in the use of antioxidants and to further thoroughly investigate the mechanisms of each substance in this pathological condition.

Some fatty acids, including polyunsaturated ones, can also be considered antioxidant compounds that show a protective effect in hemorrhagic shock. In particular, 4-phenylbutyrate had a beneficial effect on traumatic hemorrhagic shock, including improved animal survival and protection of organ function. The use of 4-phenylbutyrate improved hemodynamic parameters, reduced lactate levels, and markers of liver and kidney damage. This substance significantly increased mitochondrial function in both the liver and kidney and dilated the superior mesenteric arteries. These beneficial effects of 4-phenylbutyrate in traumatic hemorrhagic shock are probably due to its action on the vasculature by attenuating oxidative stress and the mPTP pore. Although various mechanisms of action of 4-phenylbutyrate in ammonia degradation, suppression of endoplasmic reticulum stress, and inhibition of histone deacetylase have been proposed, antioxidant activity is considered the main mechanism of protection [111]. Polyunsaturated fatty acids improved lipid oxidation in the liver and increased PPAR-α expression in mice exposed to hemorrhagic shock, which was achieved by activating the peroxisome proliferator-activated receptor-α pathway [112]. The use of alpha-lipoic acid has been shown to be effective against vascular hyperpermeability caused by hemorrhagic shock. Due to its antioxidant and anti-apoptotic effects, α-lipoic acid reduced vascular hyperpermeability, the formation of ROS, and the activity of caspase 3. Moreover, α-lipoic acid also has positive effects on the mitochondria: its application prevented depolarization of the mitochondrial membrane and the release of cytochrome c [113].

Maintaining normal levels of reducing equivalents is also possible by using hydrogen sulfide (H_2_S) or its donors [114]. H_2_S is able to maintain normal mitochondrial function by protecting membrane phospholipids from oxidation [115] and maintaining the activity of the electron transport chain, but it is necessary to ensure the operation of the Krebs cycle, i.e., adequate utilization of its intermediates, such as succinate [116]. In experimental modeling of hemorrhagic shock, administration of H_2_S in the form of NaHS prior to resuscitation had a beneficial effect on general hemodynamic parameters, and cardiac, pulmonary, and hepatic status [117,118,119,120,121]. In addition, the administration of Na_2_S reduced mortality, improved the state of various organs, and reduced the negative effects of hemorrhage on them [122]. However, the use of high doses of the mitochondrial target AP39, which is an H_2_S donor, resulted in lower mean arterial pressure, higher norepinephrine demand, and higher mortality after hemorrhage, despite attenuation of systemic inflammation in lung tissue [116,123]. On the other hand, during hypoxia, excessive accumulation of endogenous H_2_S may occur in tissues, which can negatively affect cell function due to binding to the catalytic site of cytochrome oxidase leading to its inhibition, deterioration of electron transfer in the mitochondrial respiratory chain, induction of oxidative stress, and cell death [124]. Therefore, the use of H_2_S and its donors is more appropriate after the restoration of adequate blood and oxygen supply and the onset of normal mitochondrial respiratory chain function. On the contrary, the reduction in endogenous H_2_S generation by inhibiting the enzymes cystathionine-γ-lyase and cystathionine-β-synthetase before or after the induction of hemorrhagic shock has a positive effect on the state of the organs [125,126]. Thus, the use of H_2_S, its donors, and inhibitors of its generation are therefore associated with certain limitations and should be carried out strictly in the appropriate phases of injury in order to achieve a positive therapeutic effect.

#### 3.3.5. Reduction in Cellular Apoptosis

One of the therapeutic strategies for hemorrhagic shock is the suppression of the mitochondrial apoptotic pathway. Substances that can inhibit mPTP and thereby protect cells from oxidative stress and apoptosis, such as cyclosporine A [127], improve liver and kidney function by increasing tissue perfusion after traumatic hemorrhagic shock [128]. Another substance with a similar effect is a novel nanocrystalline resuscitation fluid based on mitochondria-targeting polymer embodied malic acid (TPP@PAMAM-MR or TPP-MR). TPP-MR has been shown not only to have an antioxidant effect but also to reduce the extent of cell death, particularly ferroptosis, after hemorrhagic shock. As a result of TPP-MR therapy, the expression of the ferroptosis-associated proteins ACSL4 and COX2 in cardiomyocytes decreased, and the level of glutathione peroxidase 4, which protects cells from oxidative stress, increased [129]. In a model of cardiac injury due to hemorrhagic shock, proteoglycan lubricin therapy was shown to be effective in suppressing the activation of pyroptosis and oxidative stress [130]. Ulinastatin is thought to inhibit endothelial cell apoptosis activated in hemorrhagic shock. In vitro, ulinastatin inhibits the release of cytochrome c and the activation of caspase-3 in vascular endothelial cells, which helps to restore the potential on the inner mitochondrial membrane and reduce the amount of ROS [131].

#### 3.3.6. Other Therapeutic Approaches Affecting Mitochondria

A separate group of potential therapeutics comprises substances that influence mitochondrial fragmentation and the utilization of damaged organelles (mitophagy). The use of mitochondrial fission inhibitor-1 under conditions of hemorrhagic shock resulted in inhibition of apoptosis and mitophagy and a reduction in the accumulation of ROS [132], which was associated with a decrease in mitochondrial reticulum fragmentation and a reduction in mitochondrial dysfunction [133]. Beneficial effects of mitophagy activation have been shown with the use of bergapten, a bioactive coumarin compound that attenuates hemorrhagic shock-induced multi-organ damage and reduces NLRP3 inflammasome activation, pyroptosis, and proinflammatory cytokine production in the kidney [134]. The use of sevoflurane, which exerts its effect via activation of SIRT1, is being considered as a potential therapeutic strategy. Postconditioning with sevoflurane in in vitro and in vivo models prevented the loss of SIRT1, which plays a role in maintaining mitochondrial function and contributed to the mitigation of oxidative stress and an increase in ATP production. In addition, sevoflurane prevented mitochondrial swelling, mitochondrial cristae destruction, and vacuolization, and also activated autophagy, which was reduced as a result of hemorrhagic shock [135]. Remote ischemic preconditioning contributes to organ protection from damage by exerting its effects via parkin-dependent mitophagy.

Indeed, remote ischemic preconditioning has been shown to have a hepatoprotective effect, particularly by promoting mitophagy [136].

One of the possible aspects of the damage that develops in hemorrhagic shock is damage to the mitochondrial membrane by lysosomal enzymes. In this context, the use of urinary trypsin inhibitor (UTI), which can inhibit lysosomal enzymes and free radical production, is being considered as a possible therapeutic strategy. The administration of UTI led to a significant improvement in cardiac function after reperfusion, resulting in higher ATP and creatine phosphate levels in the myocardium, a decrease in lactate levels and the lactate/pyruvate ratio, indicating an improvement in aerobic metabolism and an increase in the NAD^+^/NADH ratio in the mitochondria [30].

The use of arginine vasopressin is suggested as a potential therapy for the prevention of hemorrhagic shock consequences such as cardiovascular collapse. In a model of decompensated hemorrhagic shock in vivo, resuscitation with arginine vasopressin was shown to improve mitochondrial function by increasing Complex I- and Complex II-dependent respiratory capacity and electron transport from Complex I to Complex III and reducing oxidative stress in the kidneys [137]. Another substance that protects endothelial cells is sphingosine-1-phosphate, which maintains the transmembrane potential of mitochondria in vascular endothelial cells [138]. A similar effect on the endothelium is exerted by p-hydroxyphenylpyruvate, which functions both as an antioxidant and as an energy substrate [139].

## 4. Conclusions

The basis for the negative and life-threatening consequences of hemorrhagic shock is multiple organ failure, which occurs as a result of ischemia of most organs. Eliminating the causes of poor blood supply (restoring circulating volume, normalizing the coagulation, and replenishing oxygen-carrying systems) cannot completely reverse the tissue damage, as hemorrhagic shock is accompanied by a cascade of pathological changes in cell physiology, leading to inefficient energy production, oxidative stress, and apoptotic death. Therefore, the right strategy to treat hemorrhagic shock and associated multiple organ failure is a combination therapy with drugs acting on different targets. Promising drugs that have been investigated in clinical and preclinical studies are antioxidants, including those targeted to the mitochondria, mitochondrial pore inhibitors, and apoptosis inhibitors. However, researchers have the highest hopes for drugs that normalize cellular energy metabolism. These may be substrates and intermediates of oxidative phosphorylation, NAD^+^ and its precursors, and activators of the PDC and the tricarboxylic acid cycle, as well as alternative substrates of oxidative phosphorylation such as ketone bodies. At the same time, the use of these agents requires a detailed understanding of the dynamics of the development of the bioenergetic crisis in hemorrhagic shock, as the effect of many of these compounds can change from positive to negative depending on the stage of the pathology, as shown, for example, with succinate and H_2_S.
